# Patient Choice and Willingness Toward Gatekeepers as First-Contact Medical Institutions in Chinese Tiered Healthcare Delivery System: A Cross-Sectional Study

**DOI:** 10.3389/fpubh.2021.665282

**Published:** 2021-06-23

**Authors:** Xia Li, Liang Zhang, Zhong Li, Wenxi Tang

**Affiliations:** ^1^School of International Pharmaceutical Business, China Pharmaceutical University, Nanjing, China; ^2^Center for Pharmacoeconomics and Outcomes Research of China Pharmaceutical University, Nanjing, China; ^3^School of Medicine and Health Management, Tongji Medical College of Huazhong University of Science and Technology, Wuhan, China; ^4^Research Center for Rural Health Services, Hubei Province Key Research Institute of Humanities and Social Sciences, Wuhan, China

**Keywords:** patient choice, patient willingness, gatekeeper, primary care institutions, first contact, tiered health care delivery system, influencing factors

## Abstract

**Introduction:** Gatekeeping mechanism of primary care institutions (PCIs) is essential in promoting tiered healthcare delivery system in China. However, patients seeking for higher-level institutions instead of gatekeepers as their first contact has persisted in the past decade. This study aims to explain patients' choice and willingness and to provide potential solutions.

**Methods:** A survey was conducted among residents who had received medical care within the previous 14 days. Patients' choice and willingness of PCIs for first contact together with influencing factors were analyzed using binary logistic regression.

**Results:** Of 728 sampled patients in Hubei, 55.22% chose PCIs for first contact. Patients who are older, less educated, with lower family income, not living near non-PCIs, with better self-perceived health status, only buying medicines, and living in rural instead of urban area had significantly higher probability of choosing PCIs. As of willingness, over 90% of the patients inclined to have the same choice for their first contact under similar health conditions. Service capability was the primary reason limiting patients' choice of PCIs.

**Conclusions:** The gatekeeper system did not achieve its goal which was 70% of PCIs among all kinds of institutions for first contact. Future measures should aim to improve gate-keepers' capability.

## Introduction

The healthcare delivery system in China is hospital-centric and fragmented: primary care institutions (PCIs) were not trusted by public for its poor quality of care and hospitals kept expanding to serve more patients (1), and there is little coordination of care among different tiers of healthcare providers (2), instead they even compete for patients and hold onto patients when they should be referred elsewhere (3). This medical chaos caused an inefficient healthcare delivery system, as well as prohibitive medical costs and widespread public discontent. To solve this chaos, in 2009, China launched a comprehensive health reform. For the first step of the reform (2009–2011), Chinese government emphasized strengthening infrastructure of PCIs, then for the second step (2012 onwards) a reform of healthcare delivery system was prioritized (4), aiming to limit specialty care access from high-level hospitals and help systems reduce overuse of inappropriate care by having PCIs perform gatekeeping functions.

In 2017, the State Council issued guidelines for initiating tiered healthcare delivery system (TDS) to enhance capability of PCIs and vertical integration among healthcare institutions in different tiers to facilitate gatekeeping function of PCIs (5). Measures of TDS include redefining facility roles, especially hospitals, within a vertical integrated network, developing formalized facility networks and establishing provider-to-provider relationships through technical assistance and skill building. One of the major goals of this policy was to promote the percentage of patients who choose PCIs for their first contact to 70% among all available kinds of medical institution.

However, to date the implementation of the system has been unsatisfactory. The share of outpatient visits at PCIs (71% in 2005, 57% in 2018) kept decreasing relative to those treated at hospitals (26% in 2005, 40% in 2018) (6). PCIs did not become more popular under the reform, and the flow of patients remained in chaos and brought about descending efficiency in healthcare delivery system (1).

To analyze the cause of failure of redirecting patients' first contact at PCIs and find feasible solutions, it is essential to understand factors influencing their choice of institutions at the first point of contact. Existing studies on the factors influencing the selection of healthcare institution for first contact have focused on individuals' willingness or attitude to go to PCIs (7–10). Studies on the reported actual behavior of people with medical treatment needs are lacking. Besides, no studies have investigated patients' first-contact willingness for medical institutions after their experience in first-contact medical institutions. Therefore, this study aims to explore the factors affecting the patients' actual choice of a medical institution for first contact, as well as first-contact willingness for medical institutions if experiencing a similar illness in the future. We use the findings of the study to provide feasible suggestions for promoting the normal function of TDS.

## Materials and Methods

The study was part of the background research designed and implemented by the Research Center for Rural Health Services, Key Research Institute of Humanities and Social Sciences at Huazhong University of Science and Technology, aiming to understand the health service needs of Chinese residents and the factors influencing these needs. The research protocol was approved by the Ethics Committee of Tongji Medical College of Huazhong University of Science and Technology (IRB No. S459, 2018). With the approval of this committee, written informed consent was obtained from respondents.

### Research Setting

After comprehensive consideration of economic, medical resources, and medical accessibility, we chose Hubei, a central province as the sample area. In 2018, per capita disposable income (US$3,947), the number of licensed physicians per 1,000 people and hospital beds per 1,000 people (2.1 and 6.65) of Hubei ranked middle-upper level (12, 14, and 8) among 31 provinces in mainland China. On the other hand, like most central provinces in China, the landforms of Hubei consist of mountains (56%), hills (24%), and plain lake areas (20%) (11), and convenient transportation enables both rural and urban residents in Hubei to have access to general or specialist medical services. Following State Council guidelines, Hubei government introduced goals and measures for TDS in June 2017. The main goal was to build up at least one effective vertical network in each city of Hubei by the end of 2017, and measures included defining responsibilities, rights, and duties of network members, integration of resources (medical techniques, medical personnel, information of patients), and establishing dual referral within networks (12). As of 2018, Hubei had already built up 626 networks covering 133 tertiary hospitals, 486 Secondary Hospitals, and 1,598 PCIs. However, no available evidence showed the effects of policy implementation on promoting gate-keeper function of PCIs.

Among 12 cities and one autonomous prefecture in Hubei, we selected Yichang as our study case. Yichang had a permanent resident population of 4,169,200 in 2018 with a moderate level of economic development. In the establishment of TDS, Yichang has put efforts on establishing dual referral system, increasing insurance reimbursement rate for PCIs, promoting family doctor system, and building information platform among institution of different tiers to facilitate first contact at PCIs and integrated clinical pathways (13). More specifically, for example, for referral system, an online referral information platform was established among 774 public healthcare institutions in 2014. As to increase reimbursement for PCIs, insured patients in Yichang diagnosed and treated in PCIs can enjoy lower deductibles and higher reimbursement rates. To promote family doctors, Yichang has implemented measures including government subsidies for health services provided by family doctors, lower prices for services, technical support from higher-level hospitals, and performance reward.

We randomly selected one urban area (X) and one rural area (D) in Yichang. Area X has a total area of 58.97 km^2^ and a permanent population of 394,200. Urban residents account for 97.94% of the total population, and per capita disposable income in 2018 is US$5,863. Area D has a total area of 2,159 km^2^, 20% of which is arable land. The area's permanent population is 469,300, with the urban population accounting for only 51.16%, and the per capita disposable income in 2018 is US$4,073. In terms of medical resources, area X has eight public hospitals and 20 PCIs (three community health service centers, eight community health service stations, and nine village clinics). Area D has three public hospitals and 155 PCIs (seven township hospitals, three street hospitals, and 145 village clinics). Area D and area X differ in terms of medical resources, with area D having fewer large public hospitals but more PCIs compared with area X.

### Study Participants and Sampling

The study participants were individuals who had received services from a medical institution in 14 days prior to the administration of the questionnaire survey. Five urban/rural communities were randomly selected in both Area X and Area D. A total of 42 households were randomly selected in each of these communities. A total of 210 urban households and 210 rural households were selected for the study sample. The average number of residences per households was 2.52.

### Questionnaire Design

The questionnaire was designed by Huazhong University of Science and Technology based on standard National Health Service Survey, published literatures, and opinions from several experts. For questionnaire validation, a presurvey of 30 respondents was conducted to test its external and internal validity. Amendments were made based on the feedback received. The final version of questionnaire contained four parts of questions: basic information of household and individual, health-related quality of life and health behaviors, uses, and demand of health services. The survey was conducted in August 2018. Based on our study design, only part of the survey questions was included as variables.

### Variables

The research outcomes were the first-contact selection and willingness of a medical institution in a similar illness. Institutions were categorized as PCIs (community health service centers in urban areas, village clinics and township street hospitals in rural areas) or non-PCIs (county/city/district health institutions, municipal health institutions, and provincial- and higher-level health institutions). We identified eight types of potential influencing factors based on published studies (14–18): (1) demographic factors: gender and age; (2) socioeconomic factors: education level, employment status, marital status, annual family income, and medical insurance; (3) accessibility of medical resources: nearest medical service provider and transportation time to the nearest medical institution; (4) health-related factors: self-perceived severity of illness and concurrence of chronic illness; (5) aims of service use this time: buying medicine, outpatient or inpatient visit; (6) residence: urban or rural area; and (7) other policy factors: registration with a family doctor or not.

### Quality Control

Before the survey, community investigators who administered the questionnaire were trained, and unified coding rules for the questionnaire and implementation steps for the survey were explained in detail. Before investigation, the purpose and significance of the investigation were explained to the respondents using a unified instruction. For low educated or illiterate respondents, investigators asked them questions and questionnaire were filled out by investigators on their behalf. Promise was made to keep the information confidential and use it only for research. With the approval of the ethics committee, written informed consent was obtained from respondents. Validity of questionnaire was checked after questionnaire collection. Questionnaires with a response rate of over 85% and no logical errors are considered valid. Questionnaires with the same answer of all items, contradictory answers, and missing or wrong answer were excluded. For data entry, Epidata 3.1 software was used to create a database, and the accuracy of these data was checked to ensure the quality of the data input.

### Statistical Analysis

The distribution of sample characteristics was statistically described. The chi-square test was used to assess the statistical significance of differences in the measured characteristics between sample populations, with *p* < 0.05 set as the two-sided significance level. The first-contact choice was analyzed using binomial logistic regression analysis (non-PCI = 0, PCI = 1). A binomial logistic regression analysis was also performed with the dependent variable of first-contact willingness (non-PCI = 0, PCI = 1). Previous studies have showed that the experience or satisfaction of PCIs were significantly associated with willingness to a future contact (15, 19). Therefore, the first-contact choice and independent variables that were significant (*p* < 0.05) in the previous regression model were included as the independent variables in this regression analysis for willingness of first contact. SPSS, version 22.0 was used for all data analyses.

## Results

A total of 854 patients were investigated, and 728 effective questionnaires were collected (effective recovery rate: 85.25%). Table 1 presents the top 10 illnesses for which participants contact PCIs or non-PCIs.

**Table 1:** Top 10 illnesses for which participants received medical services.

**Non-PCIs**	**Proportion**	**PCIs**	**Proportion**
Hypertension	11.08%	Hypertension	30.58%
Heart diseases	9.85%	Disc disease	9.02%
Disc disease	8.31%	Common cold	8.02%
Cancer	5.83%	Heart diseases	6.52%
Diabetes mellitus	5.23%	Diabetes mellitus	4.76%
Cerebrovascular disease	4.31%	Endocrine, nutritional, metabolic, and immune diseases	4.51%
Acute and chronic gastroenteritis	3.38%	Motor system disease	4.01%
Urethral calculus	3.38%	Cerebrovascular disease	3.01%
Common cold	3.08%	Acute and chronic gastroenteritis	2.51%

Table 2 presents the baseline characteristics of participants by first-contact choice. Of the 728 participants, 402 (55.22%) had selected PCIs for their first contact. Compared with the patients choosing non-PCIs, the patients choosing PCIs had significantly higher percentages of individuals between 46 and 65 years old (PCIs: 58.90%; non-PCIs: 49.85%), with less than a junior high school education level (PCIs: 89.65%; non-PCIs: 72.30%), employed (PCIs: 68.37%; non-PCIs: 53.55%), with low annual family income ( ≤ 30,000 Chinese yuan) (PCIs: 64.93%; non-PCIs: 41.36%), with PCIs as their nearest medical service provider (PCIs: 94.53%; non-PCIs: 74.23%), with not severe or average self-perceived illness (PCIs: 58.19%; non-PCIs: 44.94%), those who used services of buying medicine or outpatient (PCIs:95.77%; non-PCIs: 72.08%), rural residents (PCIs: 93.53%; non-PCIs: 69.63%), and those who were registered with a family doctor (PCIs: 43.77%; non-PCIs: 34.73%). Also, patients with a maximum of 10 min of travel time to the nearest medical institution made up significantly lower percentages of the group choosing PCIs than of the group choosing non-PCIs (PCIs: 79.45%; non-PCIs: 88.65%). However, no significant differences were found between those choosing PCIs and those choosing non-PCIs for the first contact in terms of gender, marital status, medical insurance, or presence of chronic disease.

**Table 2:** Demographic characteristics of participants by first-contact choice.

**Variable**	**PCIs**	**Non-PCIs**
	**[*n* = 402 (%)]**	**[*n* = 326 (%)]**
**Gender**		
Male	179 (44.86)	157 (48.31)
Female	220 (55.14)	168 (51.69)
Age (years)^**^		
0–45	28 (7.02)	55 (16.92)
46–65	235 (58.90)	162 (49.85)
≥66	136 (34.09)	108 (33.23)
Education^**^		
Uneducated	66 (16.67)	29 (9.24)
Primary school to Junior high school	289 (72.98)	198 (63.06)
Senior high school to junior college	39 (9.85)	80 (25.48)
Bachelor degree or above	2 (0.51)	7 (2.23)
Employment status^**^		
Employed	268 (68.37)	166 (53.55)
Retired	66 (16.84)	103 (33.23)
Student	2 (0.51)	3 (0.97)
Unemployed	56 (14.29)	38 (12.26)
**Marital status**		
Unmarried	12 (3.04)	23 (7.08)
Married	329 (83.29)	266 (81.85)
Divorced	2 (0.51)	2 (0.62)
Widowed	52 (13.16)	34 (10.46)
Annual family income (Chinese yuan)^**^		
≤ 30,000	261 (64.93)	134 (41.36)
30,000–100,000	117 (29.10)	145 (44.75)
≥100,000	24 (5.97)	45 (13.89)
**Medical insurance**		
No medical insurance	6 (1.52)	6 (1.85)
Basic medical insurance	337 (85.32)	272 (83.95)
Basic medical insurance and commercial medical insurance	52 (13.16)	46 (14.20)
Nearest medical service provider^**^		
Non-PCIs	2 (0.50)	16 (4.91)
PCIs	380 (94.53)	242 (74.23)
Private hospital or private clinic	2 (0.50)	2 (0.61)
Pharmacies and others	18 (4.48)	66 (20.25)
Time to nearest medical institution^*^		
1–10 min	317 (79.45)	289 (88.65)
11–20 min	58 (14.54)	28 (8.59)
≥21 min	24 (6.02)	9 (2.76)
Self-perceived disease severity^*^		
Not severe at all or not too severe	66 (16.46)	41 (12.58)
Average	174 (41.73)	111 (32.36)
More severe	164 (40.90)	106 (32.52)
Very severe	17 (4.08)	22 (6.41)
**Chronic disease or not**		
Yes	314 (78.30)	234 (72.22)
No	87 (21.70)	90 (27.78)
Utilization of health services^**^		
Buying medicines	248 (61.69)	134 (41.10)
Outpatient	137 (34.08)	101 (30.98)
Inpatient	17 (4.23)	91 (27.91)
Place of residence^**^		
Urban area	26 (6.47)	99 (30.37)
Rural area	376 (93.53)	227 (69.63)
Register a family doctor or not^**^		
Yes	172 (43.77)	108 (34.73)
No	145 (36.90)	161 (51.77)
Not know	76 (19.34)	42 (13.50)

* *p < 0.05*,

** *p < 0.001*.

Table 3 shows the results of the binomial logistic regression analysis for the factors influencing first-contact choice. Patients between 46 and 65 years old (OR = 2.638, 95% CI: 1.350–5.157) or more than 66 years old (OR = 3.412, 95% CI: 1.621–7.183), those who used outpatient service (OR = 1.569, 95% CI: 1.009–2.441), and those living in a rural area (OR = 5.379, 95% CI: 2.148–13.467) had higher odds of choosing PCIs for the first contact. Patients with high school to junior college education (OR = 0.354, 95% CI: 0.169–0.740), those with middle family income (OR = 0.540, 95% CI: 0.365–0.798), those with more severe self-perceived disease status (OR = 0.401, 95% CI: 0.227–0.706), and those who used inpatient services (OR = 0.152, 95% CI: 0.081–0.285) were more likely to choose non-PCIs for the first contact.

**Table 3:** Binomial logistic regression analysis of factors influencing first-contact choice.

**Variable**	**OR**	**95%CI**	
**Gender vs. male**			
Female	0.895	0.626	1.278
**Age (years) vs. 0–45**			
46–65	2.638^*^	1.350	5.157
≥66	3.412^**^	1.621	7.183
**Education vs. Uneducated**			
Primary school to junior high school	0.829	0.467	1.474
Senior high school to junior college	0.354^*^	0.169	0.740
Bachelor degree or above	0.860	0.138	5.375
**Employment status vs. employed**			
Retired	0.807	0.461	1.410
Student	3.522	0.279	44.423
Unemployed	0.938	0.528	1.666
**Marital status vs. unmarried**			
Married	1.588	0.368	6.853
Divorced	11.344	0.474	271.639
Widowed	1.921	0.396	9.322
**Annual family income (Chinese yuan) vs**. **≤30,000**			
30,000–100,000	0.540^*^	0.365	0.798
≥100,000	0.881	0.427	1.816
**Medical insurance vs. no medical insurance**			
Basic Medical Insurance	1.516	0.330	6.979
Basic Medical Insurance and Commercial Medical Insurance	1.114	0.228	5.451
**Nearest medical service provider vs. non-PCIs**			
PCIs	2.153	0.375	12.352
Private hospital or private clinic	61.069^*^	2.878	1295.694
Pharmacies and others	1.658	0.306	8.994
**Time to nearest institution vs. 1–10 min**			
11–20 min	1.300	0.751	2.251
≥21 min	1.642	0.674	3.999
**Self-perceived disease severity vs. not severe**			
Average	0.646	0.365	1.142
More severe	0.401^*^	0.227	0.706
Very severe	0.479	0.186	1.232
**Chronic disease or not vs. yes**			
No	0.812	0.508	1.297
**Utilization of health services vs. buying medicines**			
Outpatient	1.569^*^	1.009	2.441
Inpatient	0.152^**^	0.081	0.285
**Place of residence vs. rural area**			
Urban area	5.379^**^	2.148	13.467
**Register a family doctor or not vs. yes**			
No	0.941	0.622	1.422
Not know	1.041	0.616	1.759

* *p < 0.05*,

** *p < 0.001*.

Table 4 shows patients' first-contact choice and willingness. The sample size was reduced to 346 as some patients did not answer relative questions. Of the 346 patients, only 41.04% were more willing to go to PCIs at first point of contact. Of the patients who chose PCIs for first contact, 90.91% were more willing to go to PCIs and 9.10% were more willing to go to non-PCIs. Of the patients who chose non-PCIs for first contact, only 1.04% were more willing to go to PCIs and 98.96% were more willing to go to non-PCIs.

**Table 4:** First-contact choice and first-contact willingness.

**First-contact willingness**	**First-contact choice**	
	**PCIs**	**Non-PCIs**
	**[*n* = 154 (%)]**	**[*n* = 192 (%)]**
PCIs	140 (90.91)	2 (1.04)
Non-PCIs	14 (9.10)	190 (98.96)

Table 5 presents the results of binomial logistic regression analysis for the factors influencing first-contact willingness. The results show that for patients living in a rural area (OR = 8.939, 95% CI: 2.573–31.059), those who previously chose a PCI (OR = 979.264, 95% CI: 197.554–4854.159) are more willing to choose PCIs in similar illness. Patients with very severe self-perceived disease status (OR = 0.048, 95% CI: 0.004–0.547) are more willing to choose non-PCIs.

**Table 5:** Binomial logistic regression analysis of factors influencing first-contact willingness.

**Variable**	**OR**	**95% CI**	
**Education vs. uneducated**			
Primary school to junior high school	0.266	0.044	1.631
Senior high school to junior college	1.151	0.103	12.818
Bachelor degree or above	1.977	0.007	552.010
**Annual family income (Chinese yuan) vs**. **≤30,000**			
30,000–100,000	1.184	0.316	4.431
≥100,000	8.515	1.081	67.061
**Self-perceived disease severity vs. not severe**			
Average	1.759	0.332	9.327
More severe	0.773	0.171	3.485
Very severe	0.048^*^	0.004	0.547
**Nearest medical service provider vs. non-PCIs**			
PCIs	1.790	0.046	69.514
Private hospital or private clinic	2.782	0.001	9,483.602
Pharmacies and others	1.190	0.037	38.478
**Utilization of health services vs. outpatient**			
Inpatient	1.733	0.272	11.061
**Place of residence vs. urban area**			
Rural area	8.939^**^	2.573	31.059
**First-contact institution vs. non-PCIs**			
PCIs	979.264^**^	197.554	4,854.159

* *p < 0.05*,

** *p < 0.001*.

**Figure 1:**
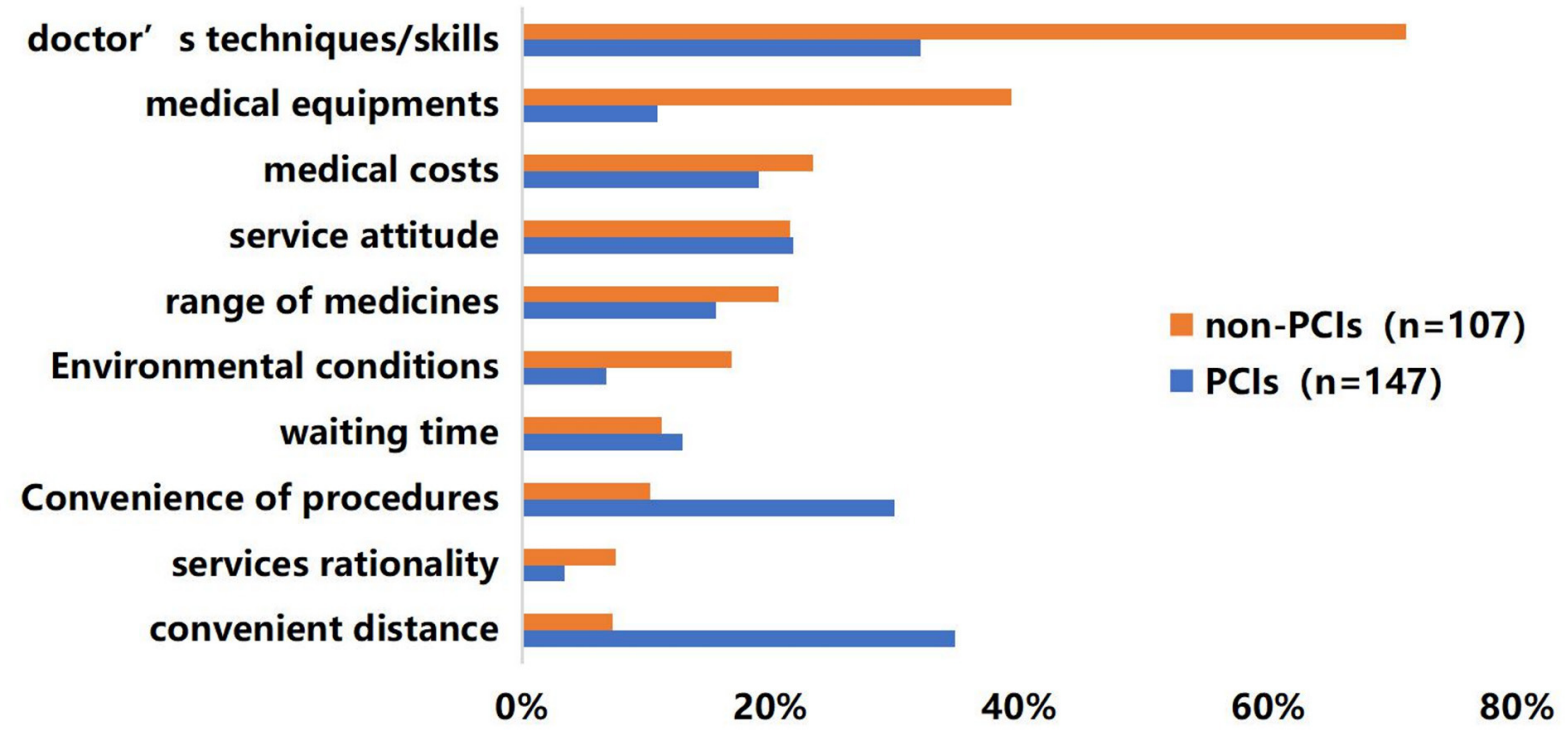
Reasons for first-contact willingness.

Figure 1 presents the reasons of why they were willing to go to PCIs or non-PCIs for first contact. Better doctor's techniques/skills (71.0%), more medical equipment (39.30%), lower medical costs (23.4%), better service attitude (21.5%), and wider range of medicines (20.6%) are the top 5 reasons for higher willingness to go to non-PCIs. For PCIs, the top 5 reasons are convenience distance (34.8%), better doctor's techniques/skills (32.0%), convenience of procedures (29.9%), better service attitude (21.8%), and lower medical costs (19.0%).

## Discussion

In this study, the percentage selecting PCIs for the first contact among participants was 55.22%, which is lower than the policy goal (≥70%) proposed by the Chinese government in 2015. We found that patients who are older, less educated, with lower family income, living nearer PCIs, with milder self-perceived disease status, buying medicine or using outpatient services (as compared to inpatient services) for their visit purpose, and living in rural area as compared with urban area are more likely to choose PCIs for their first contact.

Patients who have PCIs as nearest medical service provider were more likely to choose PCIs. Previous studies found similar results. Yong Gan et al. have reported that patients living closer to PCIs were more likely to go to PCIs for first contact (9). Jingjing Liu et al. identified distance to the nearest medical institutions as the most important factor associating with PCI preference (20). Therefore, close proximity is an advantage for PCIs such as community health centers. Given that patients with chronic diseases need dietary, lifestyle, regulatory, and pharmacological interventions (21), nearby PCIs are well-positioned to provide a cost-effective management of risk factors for chronic diseases (22).

The results show that patients who only bought medicines were more likely to choose PCIs. There are several reasons that may explain this finding. Firstly, convenient location of PCIs makes it easier to have access to pharmaceutical services. On the other hand, prices of medicines in PCIs are cheaper due to higher reimbursement in PCIs. Thus, convenience and cheaper medicines may be incentives for buying medicines in PCIs rather than non-PCIs.

Rural residents were more likely to choose PCIs than were urban residents, which is consistent with previous studies (8, 14). Haiyan Song et al. found that the health resources have impact on patients' willingness to make their first visit to PCIs (10). As is reflected in the differences in health resources between the two areas included in this study, it is a common problem in China that health resources in rural areas are lacking, compared with urban areas (23). Therefore, rural residents only passively select PCIs and their medical needs are not being met effectively (24).

Gender, medical insurance enrollment had no significantly different influence between two groups choosing PCIs or non-PCIs, which may be contradictory with existing evidence (25, 26). Besides, we found no significant impact of an individual's chronic disease status on the choice of first contact. However, according to the government guidelines, the diagnosis and treatment of chronic diseases, such as hypertension and diabetes, are considered an opportunity for a breakthrough in realizing gatekeeping function of PCIs. Consistent with this goal, Menghan Shan et al. found an increasing utilization of healthcare in PCIs and a reduction in non-PCIs with no changes in total healthcare utilization in an economically advanced city of China (27). Our finding may indicate that the system in the study areas had not been effectively promoted.

Primary healthcare has been recognized as the cornerstone of the health services system worldwide (28). Family medicine has become the dominant model for primary care in many countries (29). Family doctor system, which has been shown to play an important role in gatekeeping function of PCIs (30, 31), was not an influencing factor in our study. Da Feng et al. found that family doctors may reduce patients' tendency for unnecessary use of medical resources (32). However, according to our finding, family doctors were not playing a role in guiding healthcare seeking behaviors of patients. Well education and training of general practitioners and an effective family doctor system under the national policy are needed throughout China (33, 34).

Our findings in willingness of first-contact institutions show that patients with milder self-perceived disease status, living in rural area as compared with urban area and previously choosing a PCI for first contact as compared with non-PCIs are more willing to choose PCIs in a similar illness, which are also found in previous studies (9, 14). Besides, we also found that although most patients' first-contact willingness was consistent with their actual first-contact choice, the percentage of patients who were more willing to choose PCIs for first contact decreased compared with first-contact choice of PCIs (41.04 vs. 44.51%). Most patients believe non-PCIs rather than PCIs can better meet their medical needs.

According to the reasons for first-contact willingness, the main reason for choosing non-PCIs was doctor's techniques (71.0%). In contrast, the most important reason for choosing PCIs were convenience distance (34.8%). As to medical costs, which is much lower in PCIs thank to higher reimbursement and cheaper services; however, only 19.0% of patients considered it as their reason for choosing PCIs. To conclude, doctor's techniques and convenience may be decisive in first-contact willingness but lower medical costs in PCIs are not that attractive. Previous studies found similar results and mainly attribute these to the growth of income and the pursuit of better healthcare (35, 36).

Together, it is disappointing for policy makers and implementers to know that there is continuing decreasing choice and willingness of patients choosing PCIs as their first-contact institutions. However, the tendency is not irreversible given the Chinese government have been informed with the elephant in the room that the grassroot capability has been weakening during the past decade (34, 37–39). Similar with China, Korean government also faced up with an inefficient use of healthcare resources that about 15% of outpatient visits eligible for primary care happened in high-level hospitals (40). A cross-sectional study in Taiwan shows that the trends of bypassing primary care for treatment of common diseases decreased from 2,000, but still high for diabetes in 2017 (41). Another study in Austria found that visiting specialists were quite common and the simple presence of a general practitioner as a usual source of care was insufficient (42). In Japan, patients also can access any medical institution without referral. A study in Japan found that introduction of a gatekeeping system was necessary to reduce the incidence of referral to advanced care (43).

On the basis of the above analysis, we propose the following suggestions for potential solution for a better functioning TDS. The government of China should continue to increase financial investment, improve accessibility, and advance service capacity for PCIs (1). To meet the medication needs of patients with chronic diseases, the government should also equip PCIs with sufficient supplies of a variety of common drugs to treat these diseases. For construction of TDS specifically, efforts should be taken for a better vertical integration of urban and rural health resources (39). Potential measures could include developing distance medical services, forming exports teams to contribute to improve medical skills of staff, consultation of patients, health education, and providing smooth two-way referral in rural areas (44, 45). Besides, the number of general practitioners is still insufficient in this country given the largest population in the world and way lags behind the growth in the numbers of specialists (46). Thus, the Chinese government should pay attention to the training and incentive structures available to general practitioners, to increase the number of general practitioners and improve the level of service they offer to meet patients' medical needs.

Our study deeply explored into one factor of the TDS—the patients' choice and willingness of first-contact institution as compared with existing evidences. This study is not without limitations though. First, the sample was small and consisted only of individuals living in one city. Thus, the generalizability of the results is limited. However, from another perspective, compared with the one-fit-all approach, it is reasonable to believe that due to the differences in policies, culture, demography, environment, and so on, tailored studies in different contexts is more valid in terms of exploring behavioral causes for first-contact choice. Second, only patient-level influencing factors were considered; factors from institutional level were not investigated. Institutional characteristics such as capacity, equipment, and drug allocation are also important factors affecting patients' selection of a first-contact medical institution (25). Besides, for policy factors where only family doctor system was considered in this study, other important reform actions for TDS, like referral system, are not analyzed. In addition, even at the patient level, not all factors were included in this study. For example, previous contact history (19) and trust in PCIs (47) were not considered.

## Conclusions

This study compared first-contact choice and willingness of patients, offering a new perspective to understand factors influencing patients' selection of healthcare institution for first contact. We found that the percentage of patients choosing PCIs for the first contact fell short of the expected policy target in China's TDS, and patients who actually selected PCIs for first contact were more willing to choose non-PCIs. PCIs still need to strengthen their capacities to change patients' behavior inertia of going to large hospitals to seek medical care. Further study is still needed to develop helpful evidence on how to redirect patients' first contact to PCIs.

## Data Availability Statement

The datasets presented in this article are not readily available because the data that support the findings of this study are available from the Research Center for Rural Health Services, Key Research Institute of Humanities and Social Sciences at Huazhong University of Science and Technology but restrictions apply to the availability of these data, which were used under license for the current study, and so are not publicly available. Data are however available from the authors upon reasonable request and with permission of the Research Center for Rural Health Services, Key Research Institute of Humanities and Social Sciences at Huazhong University of Science and Technology. Requests to access the datasets should be directed to Liang Zhang, zhangliang@tjmu.edu.cn.

## Ethics Statement

The studies involving human participants were reviewed and approved by the Ethics Committee of Tongji Medical College of Huazhong University of Science and Technology (IRB No. S459,2018). Written informed consent to participate in this study was provided by the participants' legal guardian/next of kin.

## Author Contributions

WT conceptualized the research idea, developed the research design, and contributed to the manuscript revision. XL performed the data analysis and drafted the manuscript. ZL and LZ helped colleting and cleaning the data. All authors read and approved the final manuscript.

## Conflict of Interest

The authors declare that the research was conducted in the absence of any commercial or financial relationships that could be construed as a potential conflict of interest.

## Abbreviations

PCIs: primary care institutions
TDS: Tiered Healthcare Delivery System.
